# Applications of inverse opal photonic crystal hydrogels in the preparation of acid–base color-changing materials

**DOI:** 10.1039/d3ra07465j

**Published:** 2024-01-10

**Authors:** Hu Wei, Changbing Chen, Dafeng Yang

**Affiliations:** a Research Institute for National Defense Engineering of Academy of Military Science, PLA Luoyang 471023 China huwei@nuaa.edu.cn +086-18761686837; b Henan Key Laboratory of Special Protective Materials Luoyang 471023 China

## Abstract

Hydrogels are three-dimensional (3D) crosslinked network hydrophilic polymers that have structures similar to that of biological protein tissue and can quickly absorb a large amount of water. Opal photonic crystals (OPCs) are a kind of photonic band gap material formed by the periodic arrangement of 3D media, and inverse opal photonic crystals (IOPCs) are their inverse structure. Inverse opal photonic crystal hydrogels (IOPCHs) can produce corresponding visual color responses to a change in acid or alkali in an external humid environment, which has wide applications in chemical sensing, anti-counterfeiting, medical detection, intelligent display, and other fields, and the field has developed rapidly in recent years. In this paper, the research progress on fast acid–base response IOPCHs (pH-IOPCHs) is comprehensively described from the perspective of material synthesis. The technical bottleneck of enhancing the performance of acid–base-responsive IOPCHs and the current practical application limitations are summarized, and the development prospects of acid–base-responsive IOPCHs are described. These comprehensive analyses are expected to provide new ideas for solving problems in the preparation and application of pH-IOPCHs.

## Abstract 2

Hydrogels are three-dimensional (3D) crosslinked network hydrophilic polymers that have structures similar to that of biological protein tissue and can quickly absorb a large amount of water.

## Graphical abstract

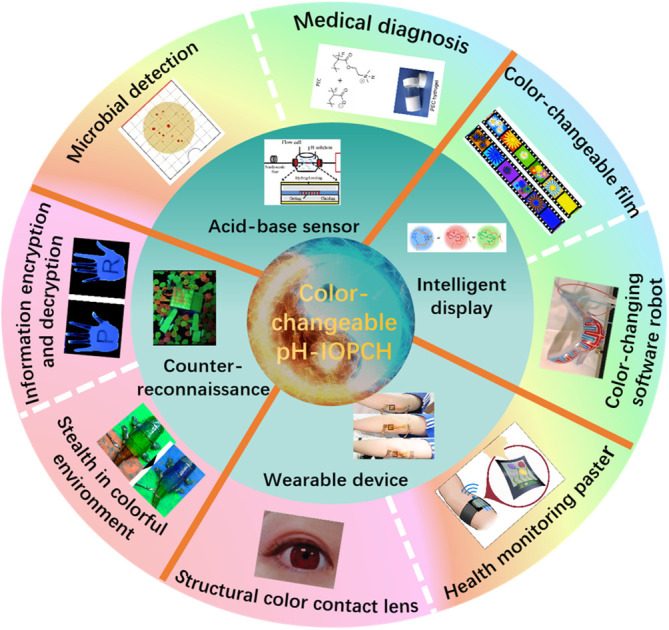

## 1. Introduction

pH-sensitive hydrogels are pH-responsive because of the large number of alkaline (such as amino, hydroxyl, amide) or acidic (such as carboxyl, sulfonic acid, *etc.*) groups in their structures.^1^ As intelligent hydrogels, pH-sensitive hydrogels have attracted much attention in many research fields in recent years, such as biology, medicine, physics, the environment, textiles, and so on.^2,3^ pH-sensitive hydrogels have become the focus of research because of their unique responsiveness to pH changes in the external environment.^4^ Based on different pH sensitivities, pH-sensitive hydrogels can be divided into swelling and shrinking types and sol–gel types.^5^ The molecular chains of these two kinds of polymers contain ionizable groups, such as carboxyl and amino groups. Taking the carboxyl group as an example, the molecular chain of this type of electrolyte or hydrogel is stretched in alkaline solutions and curled in acidic solutions. A change in the pH value of the solution can cause a further change in the molecular volume. Shao *et al.*^6^ prepared a polyelectrolyte-sensitive membrane pH sensor by using an inclined fiber grating and electrostatic self-assembly technology. With a change in the pH value of the solution, the hydrogel layer on the probe expands and contracts, which leads to a change in the optical refractive index, and the refractive wavelength shifts to red with the increase in the pH value of the solution.

A pH-IOPCH is a kind of IOPCHs with pH-sensitive characteristics. This kind of gel contains a large number of weak acidic or basic groups that are easy to hydrolyze or protonate, and they can capture or release protons according to changes in the environmental pH value, which makes the hydrogel swell or shrink because of water absorption.^7^ As a color-changing device, compare with electronic color-changing devices, IOPCHs have the advantages of simplicity, low cost, rapid and reversible response, electromagnetic interference prevention, intuitive indication effects, and so on. Compared with test paper color-changing devices, pH-IOPCHs have the advantages of all-weather automatic monitoring and long-term reuse, which have led to them being used in many color-changing systems. They have broad application prospects in intelligent devices in tissue engineering, drug delivery, fluorescent probes, microdevices, and smart skin, and can be used to produce unlabeled biochemical sensors, optical memories, and rewritable electronic paper.^8–10^ To meet the different requirements of pH detection in practical applications and reflect environmental pH with intuitive color changes, researchers have sought to develop a pH-IOPCH membrane with a simple preparation process and excellent performance (including high sensitivity, a fast response, reversible effects, automatic detection, and recycling).^11–13^

In recent years, a large number of design principles and high-performance pH-IOPCHs preparations have been reported. For example, Wang *et al.*^14^ used metal hydroxide nano-chains as templates to prepare a nano-porous polymer membrane with an adjustable pore size that was sensitive to pH and ionic strength using filtration technology; this membrane has a precisely adjustable size and has good prospects for application in the separation of ultrafine nanoparticles. However, there are few reports on the development status, synthesis principles, main technical bottlenecks, and methods of improving tunable pH-IOPCHs' performance; therefore, this paper reviews the preparation and applications of pH-IOPCHs from three different perspectives: their development status, their color-changing response principle, and methods to improve their response performance.

The structure of the paper is as follows: first, the synthesis of pH-IOPCHs is summarized based on various common materials, such as polyvinyl alcohol (PVA), polymethyl methacrylate (PMMA), polyvinylidene fluoride (PVDF), and so on, including colloidal crystal balls, colloidal crystal fibers, and colloidal particles. Then, the different discoloration principles of different IOPCHs films are analyzed. Finally, several technical bottlenecks in the preparation of pH-IOPCHs and corresponding improvement measures are summarized and listed, and future development directions are discussed.

### 1.1 Structural color of IOPCHs

After the light wave enters the surface of an IOPC, light with a specific wavelength will be prohibited from propagating and reflected, and an angle-independent light-wave diffraction effect will form on the surface, resulting in structural color, which will change with its lattice constant or refractive index.^15^ The structural color is produced by the interaction between the selective reflection of light and the unique periodic arrangement structure of photonic crystals, which has the advantages of enhanced stability, durability and tunability, depending on its unique micro/nano structure and determined by its photonic crystal arrangement periodic interval, the angle of the reflected light and the refractive index of the material.^16–18^ Therefore, the structural color can be designed by adjusting the internal micro/nano structure of IOPC.^19^ Therefore, changing the refractive index or lattice constant of an IOPC *via* external stimulation can change the structural color of photonic crystals. IOPCs are 3D interconnected porous structures formed by filling polymers into the gaps of opal photonic crystals and curing them into hydrogels, and then removing the OPC template microspheres *via* chemical reagent etching, high-temperature calcination, and other methods. Because of its certain optical band gap, it shows bright structural colors.^20^

Since the IOPCHs films have relatively different lattice distances and refractive indices, the obvious changes of unit cell volume and refractive index of the matrix confer editable color shift.^21,22^ Therefore, the internal cell structure color can be changed by using external stimulation to change the refractive index or lattice constant of IOPCHs. With the continuous development of various responsive polymers, the adjustment range of structural color is wider, and the color change mode is closer to the biological color change principle.^23,24^ For example, Wu *et al.*^25^ combined the pH-responsive luminescent pyrene tetracarboxylic acid (PTCA) into poly (*n*-isopropylacrylamide)-polyacrylamide (PNIPAM-PAAm) structure form a IOPCH film, due to asymmetric swelling and deswelling reaction, film color changes between dark brown and bright yellow in different pH environments.

Generally, nano-sized microspheres are dispersed in solvents to form monodisperse systems, and then, the OPC structure is prepared and constructed *via* self-assembly reactions in microspheres, such as evaporation, pulling, vertical deposition, and interface self-assembly. Under the action of external physical and chemical stimulation, an IOPCH with an inverse structure can produce contraction and expansion reactions. The 3D periodic-ordered porous structure inside IOPCHs has a significant ability to control the refractive index of the incident light, and the forbidden band can be directed to move to change its refractive index.^26,27^ The optical lattice constant of the IOPCs in a film can be adjusted by changing the size of the OPC ball to adjust the peak reflection position of the film.

Furthermore, inspired by the unique natural ability of chameleons to change color to reflect the surrounding environment, researchers have created responsive color-changing IOPCHs intelligent materials. IOPCHs have strong plasticity, making them very similar to biological tissue. They are bionic color-changing polymer materials with a 3D network structure that will change color after being stimulated by the outside world.^28^ Because of its network structure and the existence of hydrophilic groups in its components, a hydrogel can absorb and store a large amount of water and can maintain its color state for a long time. It can be adjusted under the physical and chemical stimulation of mechanical forces, temperature, pH, ionic solvent, electricity, illumination, magnetic field, and biological analytes, and it can then be converted into a visually recognizable color change reaction.^29^

**Fig. 1:**
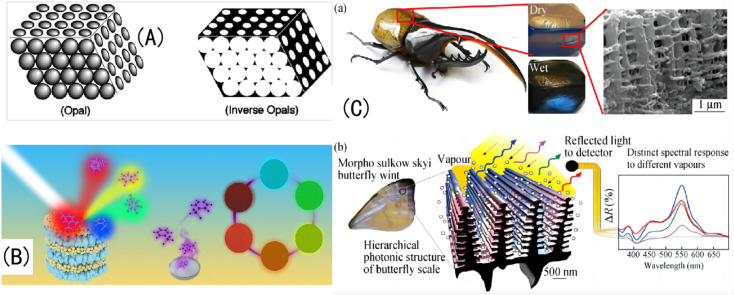
(A) Schematic diagram of OPC and IOPC structures.^32^ Reproduced from ref. 32 with permission from Wiley-Blackwell, copyright 2001. (B) Natural light is reflected by different IOPCHs films to produce different colors.^33^ Reproduced from ref. 33 with permission from Elsevier, copyright 2019. (C) (a) Photo of the exoskeleton of a humidity-responsive beetle changing from khaki to black in a high-humidity atmosphere; (b) SEM image of a stratum corneum with an IOPC structure and schematic diagram of steam humidity response discoloration of a crustacean surface based on a hierarchical IOPC structure.^34^ Reprinted with permission from ref. 34. Copyright 2010 American Institute of Physics.

Therefore, hydrogels have great potential in adaptive sensing materials, optical display devices, anti-counterfeiting, molecular switches, medical diagnosis, and color-changing stealth technology.^30^ Stimulation-responsive IOPCHs are developing toward dual-responses and multi-responses to meet the induction requirements of a complex and changeable physical and chemical environment, so it is important to scientifically and reasonably design and prepare intelligent color-changing hydrogels (Fig. 1).^31^

**Fig. 2:**
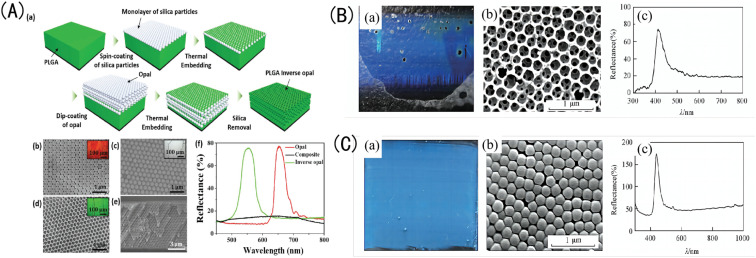
(A) (a) Schematic diagram of the preparation process for poly(lactic-*co*-glycolic acid) (PLGA) IOPCH film; SEM images of (b) the top surface of an OPC-structured film; (c) a composite OPCH film; (d) an IOPCH PLGA film, with corresponding optical crystal images as illustrations; (e) SEM images of the cross-section of a PLGA film; and (f) reflection spectra of OPC-structured film, composite OPCH film, and IOPCH film.^44^ Reproduced from ref. 44 with permission from *Adv. Mater. Interfaces*, copyright 2018. (B) (a) Photograph, (b) SEM image, and (c) corresponding Bragg reflection spectrum of silica OPC colloidal film obtained *via* TEOS hydrolysis under alkaline conditions. (C) (a) Photograph; (b) SEM image; and (c) corresponding reflection spectrum of the IOPCH film etched with an HF acid solution.^45^ Reproduced from ref. 45 with permission from Higher Education Press, copyright 2014.

The common IOPCHs preparation methods include the colloidal self-assembly method,^35^ diblock copolymer synthesis method,^36^ self-cloning method,^37^ and holographic lithography method.^38^ For example, Zhang *et al.*^39^ used silica nanoparticles and polyvinyl alcohol to atomize and co-deposit on a solid substrate and then removed the silica *via* etching to form an IOPCH film with short-range order, long-range disorder, and no iridescence effect, as shown in Fig. 2. Polymer-based flexible materials with 3D network structures can be modified with specific functional groups on the hydrogel, and the hydrogel can respond to corresponding target stimuli.^40,41^ In addition, IOPCHs films have many excellent physical and chemical properties, such as a 3D porous structure, strong mechanical expansion and contraction abilities, an easily adjustable refractive index, *etc.*, and is suitable for multi-environment production and applications.^42,43^

### 1.2 Color-changeable pH-IOPCHs films

Stimulation-responsive hydrogels are a kind of special hydrogel matrix, which can actively detect external stimuli and feedback certain responses.^46,47^ According to the types of stimuli that can be perceived, stimulus-responsive hydrogels can be divided into temperature-responsive hydrogels, light-responsive hydrogels, pH-responsive hydrogels, *etc.*^48–50^ For example, Chen *et al.*^51^ made a pH-responsive polyelectrolyte color-changing hydrogel by copolymerizing polyacrylic acid (PAA) and poly 2-(dimethylamino) ethyl methacrylate.

**Fig. 3:**
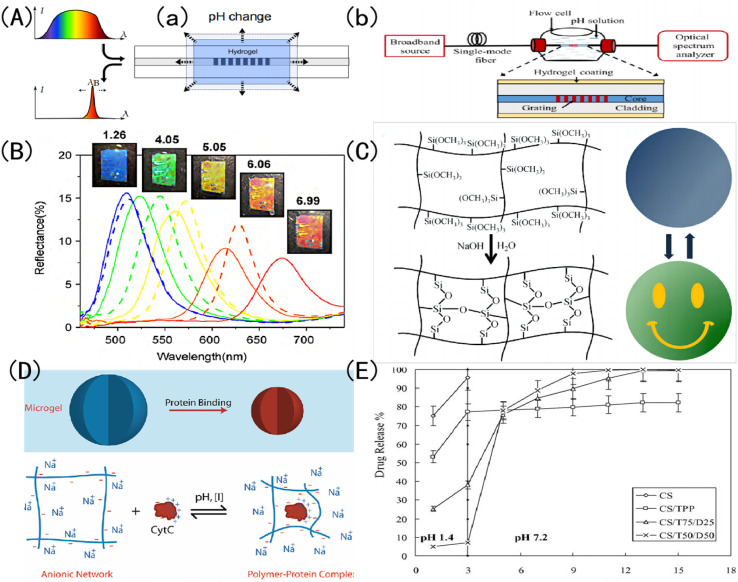
Schematic diagram of discoloration pH-IOPCHs applications in (A) (a) Bragg grating sensors;^52^ reproduced from ref. 52 with permission from IEEE, copyright 2019. (b) Long period fiber grating sensors;^53^ reproduced from ref. 53 with permission from IEEE, copyright 2016 (B): color display;^57^ reproduced from ref. 57 with permission from Elsevier, copyright 2010 (C): steganography encryption;^54^ reproduced from ref. 54 with permission from Royal Society of Chemistry, copyright 2012 (D): protein delivery;^55^ reproduced from ref. 55 with permission from American Chemical Society, copyright 2011 (E): drug release.^56^ Reproduced from ref. 56 with permission from Elsevier, copyright 2005.

Because of its high plasticity, pH response discoloration IOPCHs has a wide range of applications in sensors, color display screen, encrypted steganography, protein delivery, drug release and other fields. For example, Janting *et al.*^52^ proposed a miniature all-polymer fiber Bragg grating pH sensor element with a sensitivity of (73 ± 2) pm/pH and a response time of less than 4.5 min, as shown in Fig. 3A(a). Mishra *et al.*^53^ reported a color-changeable long-period fiber grating IOPCH sensor with pH response. When the pH value of the solution changes, the change of the volume of the hydrogel coating will lead to the change of the resonance wavelength of the transmission spectrum, thus the pH of the solution can be obtained, as shown in Fig. 3A(b). Adjust the color of the IOPCHs by changing the pH of the environment, which can be used as a color display screen, as shown in Fig. 3(B). Xuan *et al.*^54^ use polysiloxane hydrogels with different cross-linking degrees to regulate the response time difference of photonic crystals, so that the response of the low cross-linked pattern area is faster than that of the high cross-linked non-pattern region, resulting in obvious color contrast patterns in the two regions, which can be used for anti-counterfeiting steganography, dynamic identification and so on, as shown in Fig. 3(C). Smith *et al.*^55^ prepared a loose cross-linked *N*-isopropylacrylamide (NIPAm) and acrylic acid (AAc) copolymer microgel, which showed high protein loading capacity and could control protein release by regulating the pH of the environment, as shown in Fig. 3(D). Lin *et al.*^56^ prepared porous chitosan (CS) polyelectrolyte hydrogel microspheres by ionic cross-linking of sodium tripolyphosphate (Na^+^-TPP) and dextran sulfate (DS). CS/TPP/DS microspheres can transport ibuprofen from the stomach in an acidic environment to the intestines in an alkaline environment, as shown in Fig. 3(E).

At the same time, the 3D network unit in the pH-IOPCHs membranes contains a large number of polymerization monomers with hydroxyl, amide, carboxyl or amino groups, which have excellent hydrophilicity and their molecular weight and conformation can be easily controlled.^58^ In other words, the high editability nature of pH-IOPCHs membranes creates more available options for its application. Therefore, the pH-IOPCHs membranes can also be used in biomedical fields, such as biomass sensing, medical diagnosis systems, contact lenses and so on.^59^ Further, inspired by the chameleons' unique natural strategy of changing their skin color with the surrounding environment to adapt to and hide in the surrounding environment, the researchers imitated its color-changing principle, which stimulated the development of color-changing pH-IOPCHs membranes with advantages in real-time visual visualization.^60^

**Fig. 4:**
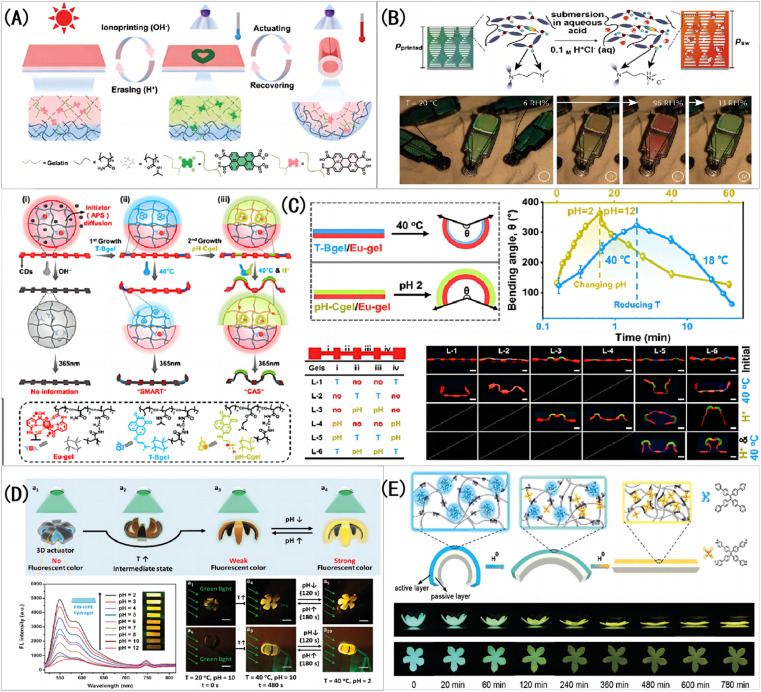
(A) A schematic diagram of the synthesis principle of PNIPAM-PAAm/PTCA color-changing pH-IOPCH actuator film.^25^ Adapted with permission reproduced from ref. 25 with permission from Wiley-VCH Verlag, Copyright 2019. (B) The color change of cholesteric liquid crystal hydrogel before and after acid treatment and its shape change with humidity.^61^ Adapted with permission reproduced from ref. 61 with permission from Wiley-VCH Verlag, Copyright 2022. (C) The composite hydrogel composed of pH-IOPCH (pH-Cgel), Eu^3+^ coordinated red hydrogel (Eu-gel) and temperature-responsive hydrogel (T-Bgel) shows editable deformation and discoloration functions.^62^ Reproduced from ref. 62 with permission from John Wiley & Sons, copyright 2023. (D) Photos of deformation and discoloration reaction of PBI-HPEI/GO-PNIPAM double-layer pH-IOPCH composite membrane with pH value change.^63^ Adapted with permission reproduced from ref. 63 with permission from Wiley-VCH Verlag, copyright 2018. (E) Photo of the color, brightness and shape of the TPE-4Py/PAS double-layer PH-IOPCH composite film changing simultaneously with pH value.^64^ Reproduced from ref. 64 with permission from Wiley-VCH Verlag, copyright 2020.

For example, Wu *et al.*^25^ prepare a PNIPAM-PAAm/PTCA pH-IOPCH membrane with color-changing property based on asymmetric expansion behavior inside the gel, as shown in Fig. 4(A). Sol *et al.*^61^ prepared a cholesteric liquid crystal pH-IOPCHs color-changing actuator, which produced asymmetric expansion characteristics after acid treatment, and reversible color and shape changes could be controlled by humidity, as shown in Fig. 4(B). Wang *et al.*^62^ synthesized a pH-IOPCH membrane capable of controlled deformation and color information encryption by copolymerizing pH responsive hydrogel (pH-Cgel), Eu^3+^ coordinated red hydrogel (Eu-gel) and temperature responsive hydrogel (T-Bgel), as shown in Fig. 4(C). Chen *et al.*^63^ prepared a double-layer hydrogel with both discoloration and deformability by combining the supramolecular assembly layer of polythyleneimide hyperbranched pertoluene diimide (PBI-HPEI) with a graphene oxide (GO)-PNIPAM layer. The color of PBI-HPEI hydrogel can be reversibly adjusted by the change of pH value, while the GO-PNIPAM layer shrinks and deforms when heated, as shown in Fig. 4(D). For example, Tang *et al.*^64^ developed a double-layer color-changing pH-IOPCH membrane composed of monomer tetrad (4-pyridyl phenyl) ethylene (TPE-4Py) and Poly (sodium acrylamide-R-4-styrene sulfonate) (PAS). Due to the aggregation of hydrophobic TPE-4Py, it shows blue fluorescence in neutral pH environment. When the pH is reduced, the TPE-4Py in the hydrogel is protonated and dissolved, causing the film to turn yellow in color and become less bright, as shown in Fig. 4(E).

## 2. Preparation of color-changeable pH-IOPCHs film

To date, integrating photonic crystal nanoparticles into self-assembled block copolymers or crystal colloid arrays (CCAs) in polymers has been a common method of constructing pH-IOPCHs.^65^ For example, Asher *et al.*^66^ designed a mechanically responsive photonic crystal hydrogel color-changing film by embedding CCAs of polystyrene (PS) in the copolymer of *N*-vinylpyrrolidone and acrylamide (AM) for the first time, which pioneered this field. Thereafter, OPCs were combined with stimulus-responsive hydrogels, and a series of pH-and ionic-strength-sensitive polyacrylamide (PAM) color-changing IOPCH sensing materials were prepared using the electrostatic self-assembly method; these can be used for the visualization detection of temperature, pH, metal ions, *etc.*, in the environment.^67–70^ After years of development, IOPCHs have been prepared that can adjust the color-changing response of the cell volumes of pores in polymers to physical and chemical stimuli such as ion concentration, pH value, temperature, and solvent type, and the response speed and adjustment accuracy have also been greatly improved.^71,72^

### 2.1 Three-step template method

**Fig. 5:**
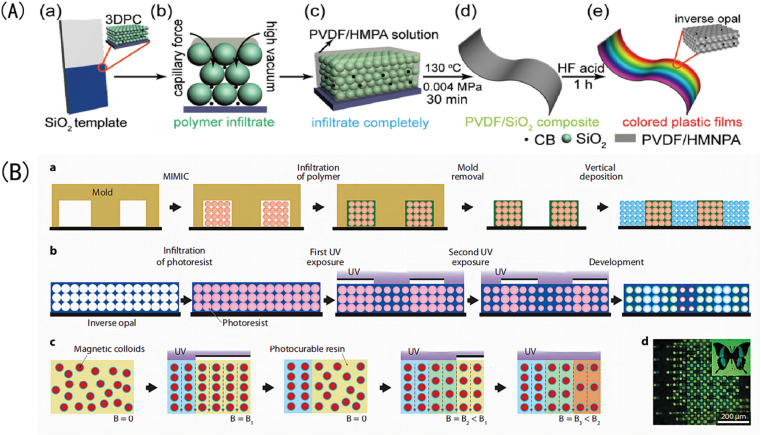
Schematic diagram of (A) preparation of color polyvinylidene fluoride (PVDF) inverse opal structure film using the three-step method.^76^ Reproduced from ref. 76 with permission from John Wiley & Sons, copyright 2018. (B) Patterning of photonic crystals: (a) IOPCHs films were prepared *via* direct deposition, (b) exposing the porous IOPCHs films filled with SU-8 photoresist at 0° and 90° twice under ultraviolet light through a linear photomask, (c) then, superparamagnetic particles are arranged in chains under an external magnetic field, and the structure is partially fixed *via* exposure to a selective ultraviolet lamp to form a chain pattern, (d) thus forming a butterfly image consisting of blue and yellow pixels.^77^ Reproduced from ref. 77 with permission from Springer Nature, copyright 2011.

Template etching is the most important method of preparing IOPCHs at present; this method can be briefly summarized in three steps.^73^ First, the nanoparticles are assembled to form OPC templates using appropriate methods. Second, an organic precursor liquid, such as a polymer monomer, organic metal salt, metal alkoxide, *etc.*, fills the gap in the template and is then cured using ultraviolet irradiation or heating polymerization. Finally, the nanoparticle template is removed to obtain the IOPCH film, as shown in Fig. 5.

The specific preparation steps of the three-step method can be briefly summarized as follows: (1) assembling OPC colloidal microspheres into a 3D template; (2) filling the gaps in the template with polymer and curing; and (3) removing the template to prepare the IOPCH film.^74^ For example, Kim *et al.*^75^ directly added an 8 wt% (mass fraction) silk fibroin solution to a PMMA template to fill it; then, they dried it at room temperature for 24 h to volatilize the solvent and removed the template to obtain an IOPCH membrane with uniform pores and structural color.

### 2.2 Two-step method

**Fig. 6:**
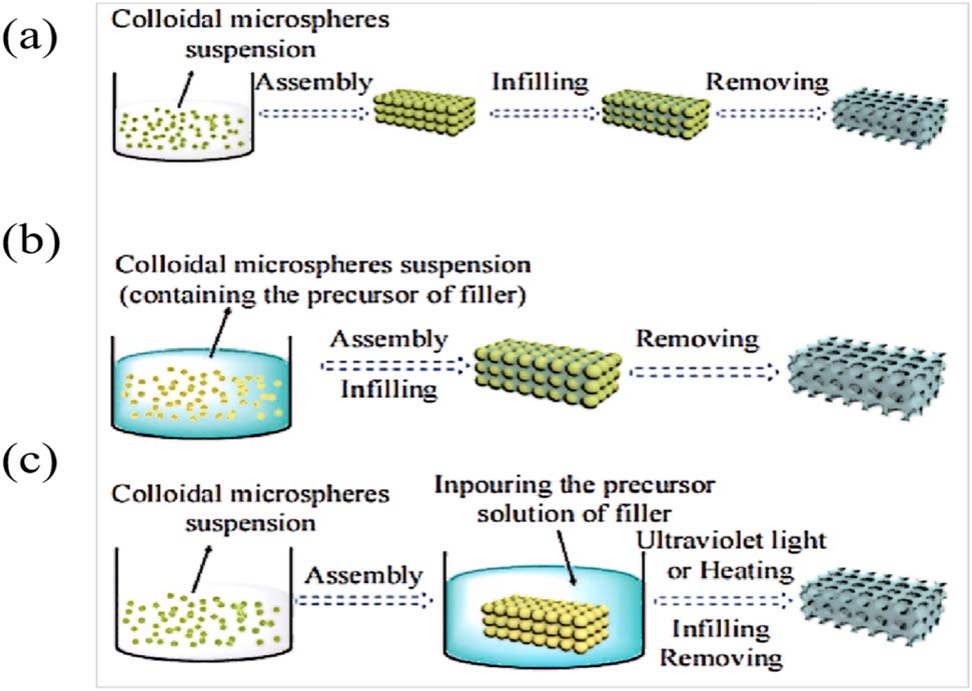
Schematic diagram of three-step (a) and two-step (b and c) preparation of an IOPCH membrane.^78^ Reproduced from ref. 78 with permission from Frontiers Media S.A., copyright 2020.

The two-step preparation of IOPCHs membranes usually combines two consecutive steps in the three-step method into one, that is, combining the template assembly and high-polymer-content membrane material filling synthesis steps or the membrane material filling synthesis and the template removal steps. Compared with the three-step method, the two-step method is simpler, but combining the template assembly and film-forming material filling into one step requires suitable solvents. Alternatively, by using the film-forming material to fill the gap and removing the template at the same time, the film-forming material can be directly obtained by heating the precursor, as shown in Fig. 6.

### 2.3 Preparation of template

At present, there are only a few kinds of nanospheres used to prepare photonic crystals, such as SiO_2_, PS, PMMA, *etc.* Whether the assembled template is regular or not determines the uniformity of the holes in the IOPCHs films. The common template assembly methods are described in Table 1.

**Table 1:** Advantages and disadvantages of different template preparation methods

Preparation method	Technological process	Advantage	Disadvantage	References
Vertical deposition method	Puts the substrate vertically into a monodisperse colloidal microsphere assembly solution, and after the solvent evaporates, regular OPC templates are piled up on both sides of the substrate	The OPC template is uniform and easy to adjust in thickness, but it takes a long time and requires high temperature, humidity, shock-resistant equipment	The thickness of the OPC template is difficult to control, with many defects and an uneven structure	79
Spin coating method	Colloidal microspheres are dispersed on a rotating table, and after the solvent evaporates, an OPC template is formed on the substrate	The preparation process is simple, and the time consumption is short	Selecting a dispersion concentration and spin-coating parameters are required	80
Spraying method	Colloidal microsphere solution is refined into spray and superimposed on the substrate to form an OPC structure	Can quickly prepare a large-area crystal template	OPC microspheres are easy to adhere to the substrate but cannot easily fall off the substrate	81
Magnetic induction method	With the assistance of a magnetic field, an OPC template embedded with magnetic nano-chains can be prepared *via* one-step polymerization	The contrast of the refractive index is large, and the structural color is uniform and bright	Cannot exist without the magnetic field, and the preparation area is limited	82

### 2.4 Template filling

Template filling is a crucial step in preparing an IOPCH film using the three-step method, and the filling rate directly affects its performance.^83^ The commonly used filling methods are described in Table 2.

**Table 2:** Comparison of different common filling methods for preparing IOPCHs membranes templates

Preparation method	Technological process	Advantage	Disadvantage	References
Solvent evaporation method	Covering the film-forming material on the template, and evaporating the solvent to obtain a solid filled film. It can be divided into dip coating method, drop coating method and jet printing filling method	Simple operation and low requirements for equipment	It is easy to cause defects in the membrane structure. Excessive filling material will lead to non-porosity, and it will become difficult to remove the template. Insufficient filling material will lead to an uneven IOPCHs films	84
Sol–gel method	The OPC template is filled with organic solvent as a precursor, and a stable gel forms after polymerization. After removing the OPC template, the IOPCH film is obtained	The product has high purity, good uniformity, a low synthesis temperature, and it is easy to control the stoichiometric ratio and reaction conditions	There is some environmental pollution in the process of organic synthesis	85
Chemical vapor deposition method	The gas precursor penetrates the gap of the OPC template and undergoes a chemical reaction to complete the filling	The filling rate is fast, and a uniform IOPCH film can be made	The process temperature is high, and the required equipment is complex	86
Electrochemical deposition method	A film-forming material is electrochemically deposited in a gap in the OPC template, and the IOPCH film is obtained after the OPC template is removed	The membrane structure is controlled by directly adjusting the electric field intensity, the concentration of the suspension, and the amount of charge carried by the colloidal microspheres	The electric double layer on the electrode surface affects the charged colloidal microspheres to some extent, which makes the order of the obtained IOPCH membrane pores low	87

### 2.5 Removal of template

The common methods for removing photonic crystal templates are as follows: (1) the heating method, which heats the filled template to a certain temperature to make it thermally decompose; (2) the chemical dissolution and corrosion method, in which appropriate reagents are selected to dissolve or corrode the template.

For the composite OPC structure filled using the sol–gel method, heating can also concentrate the template structure and promote film growth. However, in the heat treatment process, the polymer's inverse opal structure is prone to collapse, and the template can easily melt and adhere to the film, so the heating method should be used with caution, as appropriate. For polymers that are not resistant to high temperatures, the template is often removed *via* solution corrosion. Generally, the SiO_2_ template is removed using hydrofluoric acid, PMMA can be dissolved and removed using acetone, and PS can be removed with tetrahydrofuran or toluene.^88^

The above shows that the processes of preparing IOPCHs films by stripping the photonic crystal template are similar, but the color-change principle and the influencing factors of the film's response performance are different because of the different colloidal materials used. In the following, the discoloration mechanisms of several common different types of acid–base electrochromic IOPCHs films are described, and the influencing factors of their discoloration responsiveness are summarized.

## 3. Discoloration mechanisms of different types of IOPCHs films

Based on the above, the advantages and disadvantages of several preparation methods for IOPCHs films can be compared. The discoloration mechanism can be determined by combining changes in the physical and chemical properties of the IOPCHs films in order to summarize the factors affecting its discoloration performance and the methods of improving its performance.^89^ Color-changeable pH-IOPCHs usually contain a large number of weakly acidic or alkaline molecular groups that are easily hydrolyzed or protonated. These molecular groups will ionize with changes in pH intensity in the environment and capture or release protons, such as ammonium salts, carboxyl groups, or sulfonic groups.^90^ These groups will dissociate or polymerize with the change in the environmental pH value, resulting in a change in the ion concentration inside and outside the hydrogel and the interaction force between polymer molecular groups; the physical properties, such as the porous unit cell volume and refractive index, will also change.

At the same time, these ionized groups will also destroy the hydrogen bonds between the molecular chains of the hydrogels, which will make the hydrogels swell or shrink because of water absorption, thus causing the volume to change in the porous unit cells inside the hydrogels. The shrinkage–swelling transition in hydrogels provides them with good qualities for use as pH sensors and in other fields. For example, Pabbisetti *et al.*^91^ used the hydrogel expansion with changes in pH, thus causing the movement of Bragg grating, and prepared a wavelength-tunable optical fiber sensor for pH tuning; their sensing system has a good linear shrinkage–expansion response in a pH range of 3–7, with a sensitivity of 12.16 pm/pH and good repeatability. pH-sensitive hydrogels based on biocompatible copolymers have also been widely used in drug detection systems.^92^

Li *et al.*^93^ reported on a pH sensor using single-layer photonic crystal polyelectrolyte gels. Because the protonation of pyridine groups in colloidal molecules produces weak cationic polyelectrolytes, this gel can show a rapid and remarkable swelling behavior in an acidic environment, and the difference in the pH value of the environment can be read based on the color change. Therefore, the two-dimensional (2D) polyelectrolyte photonic crystal can rapidly respond to external acid–base changes. Lee *et al.*^94^ synthesized a pH-responsive color-changing IOPCHs using the colloidal crystal template method; hydrogel films were prepared by copolymerization 2-hydroxyethyl methacrylate and acrylic acid (AA). The position of the diffraction peak shifted with a change in the pH value, and the shift amplitude could be adjusted by changing the concentration of AA. Shin *et al.*^95^ prepared a pH-responsive color-changing IOPCHs by filling the OPC template with polymer precursor, initiating photopolymerization using ultraviolet irradiation, and then removing the template. The hydrogel had a rapid response time of about 10 s; at the same time, it had high mechanical strength and environmental tolerance and it can even repeatedly display changing bright colors within half a year.

### 3.1. Principle of volume expansion discoloration

Generally, the discoloration reaction caused by the volume expansion of the hydrogel is the most widely used: amphoteric sensitive hydrogels contain both weak acidic groups and weak basic groups, so this kind of hydrogel can reduce or increase the swelling ratio when the acidity or alkalinity of the solution reaches a certain value; microscopically, this is caused by a shrinkage or expansion in the hydrogel's volume. Specifically, pH-responsive hydrogels usually contain amphoteric groups such as carboxyl groups, amino groups, sulfonic groups, and so on, which can be protonated or deprotonated according to changes in the environmental pH.^96^ With a decrease in the solution's pH, the degree of protonation increases, the ion content in the hydrogel system decreases, the repulsive force between ions decreases, the intermolecular gap decreases, and the swelling rate decreases. Microscopically, the volume of the hydrogel shrinks, and macroscopically, the diffraction peak is blueshifted, resulting in a corresponding color change response. With an increase in the pH value, the opposite change occurs. However, in a near-neutral environment, the positive and negative charges are neutralized, and the swelling rate of the hydrogel decreases.^97^

For example, Wang *et al.*^98^ prepared a semi-permeable PAA-PSS network system made of a PAA hydrogel coated with polystyrene sulfonate (PSS). When the solution's pH value is 3, it is lower than the p*K*_a_ of AA. Because the hydrophilicity of –COOH is lower than that of –COO^−^, the carboxyl groups in the network chain mainly exist in the nonionic form of –COOH; thus, the electrostatic repulsion between the charged groups is small, the hydrophilicity of PAA is weakened, and the hydrophobicity is relatively enhanced. Because of the hydrophobic interaction between the carbon skeleton and the sidechain methyl group, PAA adopts a highly compressed aggregation conformation, and the reflection peak shifts to blue. With an increase in the pH value from 3 to 8, the pH value of the solution is higher than the p*K*_a_ of PAA, and the carboxyl group gradually transforms into the ionic form –COO^−^.

**Fig. 7:**
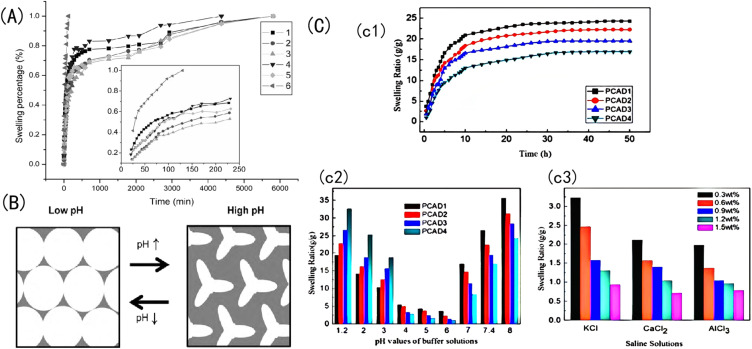
(A) Change in the swelling rate of Aam-HPMA-MA with time spent in salt solutions.^98^ Reproduced from ref. 98 with permission from Taylor and Francis Ltd., copyright 2006. (B) Schematic diagram of the changing state of the porous gel of an IOPCH membrane in a low- and high-pH-value solution environment.^95^ Reproduced from ref. 95 with permission from Elsevier, copyright 2010. (C) (c1) Swelling ratio values of CS, AA, and 2-(dimethylamino) ethyl methacrylate (DMAEMA) copolymer IOPCH membrane in deionized water; swelling kinetics in (c2) different buffer solutions and (c3) salt solutions.^102^ Reproduced from ref. 102 with permission from Royal Society of Chemistry, copyright 2013.

At this point, the hydrophilicity of the PAA is enhanced, and the electrostatic repulsion between the charged groups gradually increases. The electrostatic repulsion of –COO^−^ leads to the existence of PAA chains in an extended irregular linear conformation: the free energy of the system is minimized, the swelling ratio increases, and the reflection peak is redshifted. With the addition of PSS polyelectrolytes, the osmotic pressure of the network increased, and the osmosis and ionic electrostatic repulsion played a positive role in increasing the swelling ratio of the PAA-PSS.^99^ When the pH value is 8, the concentration of –COO^−^ in the hydrogel reaches a maximum; accordingly, the electrostatic repulsion between the molecules rises to a maximum, and the swelling ratio of the sample reaches a maximum. However, if the pH increases to 14, the concentration of –COO^−^ no longer increases, and external ions flow into the hydrogel network, which strongly increases the ion-shielding effect, decreasing the swelling ratio.^100,101^ At this point, the osmotic pressure interaction inside and outside the membrane, the mutual repulsion of the ionic strength, and the mutual entanglement between the groups do not change with the pH value, as shown in Fig. 7.

**Fig. 8:**
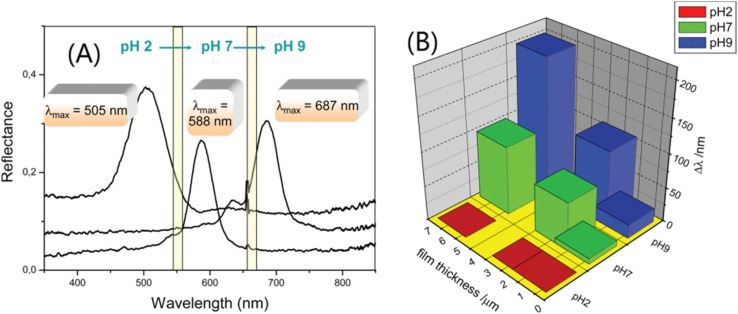
(A) Optical response curves of IOPC-structured films soaked in solutions with different pH values. (B) The relationship between Bragg peak changes (*δλ*) and the pH of IOH films with different thicknesses.^104^ Reproduced from ref. 104 with permission from American Chemical Society, copyright 2012.

The change in the Bragg peak with pH is consistent with the pH response characteristics of IOH (I representing a carbonyl). Fig. 8(A) shows that the diffraction peak of 2-hydroxyethyl methacrylate (HEMA) IOPCH films in different pH environments redshift from 505 nm at pH 2 to 687 nm at pH 9. The migration of the diffraction peak is caused by its dependence on the ionization of the carboxyl groups of IOH, which leads to the fixation of IO^−^ ions in the gel and enhances the osmotic pressure of the membrane.^103^ Therefore, with an increase in the pH value, the ionization rate of the carbonyl group increases, the gel swells, and the diffraction peak is redshifted; furthermore, with the increase in the hydrogel's thickness, the diffraction peak shifts more, as shown in Fig. 8(B).

### 3.2. Principle of copolymer discoloration

① There is an alternate-layered stacked structural membrane called a diblock copolymer that is formed by stacking two polymers with different electrolyte types. With an increase in the pH value, the conjugated segments of the hydrogel grow under the electrostatic repulsion, and the maximum absorption peak is redshifted. For example, Xia *et al.*^105^ prepared a layered stacked diblock copolymer (PS-b-P2VP) using PS and poly(2-vinylpyridine) self-assembled; and introduced AA and HEMA monomers into the partially quaternized P2VP (QP2VP) layer using the spin coating method, obtaining a pH-responsive IOPCH film. The positively charged P2VP layer showed highly reversible swelling in the acidic and alkaline pH ranges. The pH response of this type of IOPCH film can be attributed to the alternating generation of excessive positive and negative charges, and three stop bands are generated. When the pH value of the solution is 9.5, the p*K*_a_ of the protonated P2VP unit and the p*K*_a_ of the AA unit are 4.9 and 4.7, respectively. When the pH value of the solution is 3.6, the unprotonated P2VP unit is protonated, but the AA unit has no charge at this pH value.^106^

**Fig. 9:**
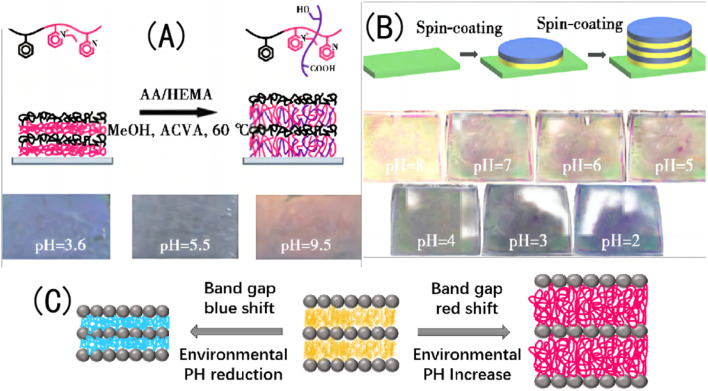
(A) Schematic diagram of molecular-crosslink-modified PS-b-QP2VP membrane prepared using the copolymerization of AA and the HEMA monomer (above), and photos of a color-changing membrane in different pH environments (below).^105^ Reproduced from ref. 105 with permission from Royal Society of Chemistry, copyright 2011. (B) Schematic diagram of pH/temperature-sensitive dual-response PS-b-QP2VP film prepared using the spin coating method (above) and photos of a color-changing film in different pH environments (below).^108^ Reproduced from ref. 108 with permission from Elsevier, copyright 2015. (C) Schematic diagram of polymer crystal colloid array transformation.

Because there are two different types of pH-sensitive polymers in the film, they have different expansion coefficients under the action of different pH values, and the relationship curve between their reflection spectra and pH displays a U shape, showing a wide band gap shift (300–900 nm). However, when an ionic AA monomer is introduced into the charged QP2VP layer, it can cause an uneven monomer distribution because of interactions between the charges. This uneven distribution of the monomer components will cause uneven expansion and contraction of the hydrogel, forming an uncontrollable complex “rainbow color” and reducing the resolution accuracy for the naked eye.^107^ At the same time, given the inherent small refractive index contrast of the diblock copolymer, the film shows a dark structural color, as shown in Fig. 9(A). The hydrogel layer formed by the copolymerization of AA and the *N*-isopropylacrylamide monomer on the silicon wafer creates the pH/temperature-sensitive IOPCH film, as shown in Fig. 9(B). Therefore, Liu *et al.*^108^ added TiO_2_, with a high refractive index, as one of the alternating layers of the layered stack using the spin coating method to improve the structural color brightness and color stability of the film. Changes in the environmental acidity and alkalinity cause an expansion and a red shift in the colloid band gap or a contraction and a blue shift in the colloid band gap, the principle of the color change of films in different pH environments is shown in Fig. 9(C).

② Graft copolymerization refers to the method of preparing IOPCHs materials *via* covalent connections between α-olefin monomers and natural polymers (such as starch, carboxymethyl cellulose, *etc.*) and their derivatives caused by hydrogen bond interactions between carbonyl groups in the polymer molecules and hydroxyl groups and amino groups of the graft molecules.^109–111^ Introducing cationic groups *via* grafting can endow hydrogels with pH responsiveness.

**Fig. 10:**
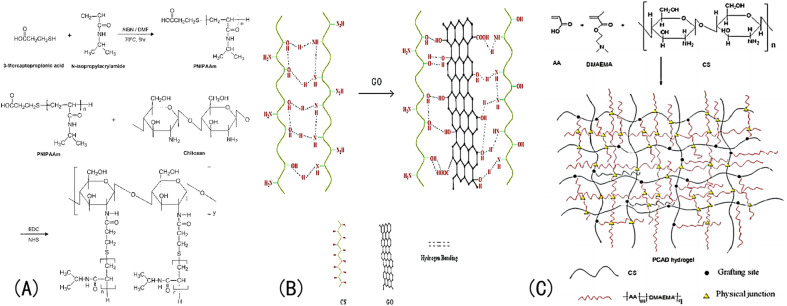
(A) Synthesis roadmap of porous PNIPAAm IOPCH membrane prepared *via* graft copolymerization.^112^ Reproduced from ref. 112 with permission from John Wiley & Sons, copyright 2004. (B) Schematic diagram of network structure of GO/CS hydrogel with high swelling rate.^114^ Reproduced from ref. 114 with permission from John Wiley & Sons, copyright 2018. (C) Preparation roadmap of CS-*co*-AA-co-DMAMEA hydrogel.^102^ Reproduced from ref. 102 with permission from Royal Society of Chemistry, copyright 2013.

For example, Lee *et al.*^112^ prepared a graft pH sensitivity hydrogels, which composed of chitosan and PNIPAm. The synthesis method for this hydrogel is shown in Fig. 10(A). The molecular structure of CS contains amine and hydroxyl groups, so a hydrogel made of CS can respond in a certain pH range in acid–base environments.^113^ Nath *et al.*^114^ introduced GO to a CS network and the swelling rate of the IOPCH membrane increased by 60–160 times, as shown in Fig. 10(B). Li *et al.*^102^ synthesized a pH-sensitive semi-interpenetrating network hydrogel P(CS-*co*-AA-co-DMAMEA) with high mechanical strength from CS, AA, and the DMAEMA monomer *via* free-radical polymerization in water, as shown in Fig. 10(C).

**Fig. 11:**
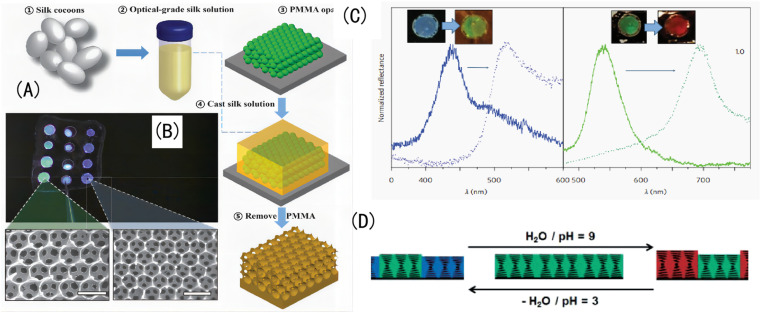
(A) (a–e) The manufacturing steps of independent silk opal. (B) Different colored IOPCH films and their corresponding SEM images. (C) The visible spectra of different colored IOPCH films.^119^ Reproduced from ref. 119 with permission from Springer Nature, copyright 2012. (D) Schematic diagram of PAA's light wavelength with pH value.^120^ Reproduced from ref. 120 with permission from Wiley-VCH Verlag, copyright 2015.

③ In addition to copolymerization, grafting, and the double-network (DN) structure, there are other structural forms and even natural amphoteric polymers, as shown in Fig. 11. For example, the manufacturing steps of independent silk opal are as follows, as shown in Fig. 11(A): obtaining optical-grade silk fibroin solution from silkworm cocoons; generating PMMA opals with diameters of 250, 320, and 500 nm on silicon substrates; infiltrating PMMA opals onto silicon wafers using the silk fibroin solution; removing the silk fibroin film from the silicon wafer after drying; and dissolving the PMMA balls in the silk fibroin film using acetone. Different colored IOPCH films and their corresponding SEM images are shown in Fig. 11(B). The color of the relevant structure under biological tissue and the reflection curve in its corresponding visible spectrum are shown in Fig. 11(C). Schematic diagram of a cholesteric liquid crystal network PAA polymer network changing its incident light wavelength along with its pH value is shown in Fig. 11(D).

Mahdavinia *et al.*^115^ prepared a semi-interpenetrating polymer network IOPCH composed of CS and PAM and found that it shrank and swelled several times in a solution ranging from pH 2 to pH 10. Other natural polymers have both amino and carboxyl groups; the molecular chain of gelatin contains hydrophilic groups such as –NH_2_, –OH, and –COOH, which can be used as matrix materials for hydrogels.^116^ Pettignano *et al.*^117^ synthesized a pH-responsive biomass hydrogel with a self-repairing ability by constructing a dynamic covalent bond between gelatin and sodium alginate oxide. After soaking in NaOH solution at pH 13, the hydrogel still had a self-repairing ability, but after soaking in an HCl solution at pH 1.36, the hydrogel lost its self-repairing ability, which confirmed the Schiff base bond (–N=CH–) pair between the gelatin and sodium alginate oxide.

Taking poly(Schiff bases) (PSBs) as an example, Ji *et al.*^118^ synthesized a PSB film containing *N*-(4-aminophenyl)-*N*-(*p*-tolyl) benzene-1,4-dialdehyde *via* polycondensation. At first, the film was light yellow, with absorption peaks at 332 nm and 400 nm. With the introduction of hydrochloric acid vapor, the absorption peak shifted to 537 nm. These changes can be attributed to the protonation of N, where the N in the C=N double bond is protonated by acid and π electrons in the donor–absorber structure of the polymer are separated, which leads to overlapping electron clouds, a reduction in the energy gap, and the red color of the film. When PSB solutions turn purple, the color change is not obvious after adding acid, and the color of PSBs changes from purple to light yellow after introducing ammonia. This is because protonated N^+^ is neutralized in alkali as it obtains electrons, and its color becomes the original color.

### 3.3. Principle of hole parameters discoloration

Because an IOPCH membrane is composed of porous spaces connected by hydrogels, solutes can diffuse through both pore spaces and the hydrogel networks. When the IOPCH membrane is in an acidic environment, it can be known from above, the hydrogel shrinks obviously, the volume decreases, the pore spaces increase relatively and are filled with free solution. Because the pore space is dominant and highly interconnected, most solution ions generally diffuse rapidly through the pores and then penetrate the hydrogel, which leads to the acceleration of the response time of ion diffusion (even just need 8 s).^121^

In alkaline environments, hydrogels expand in volume, occupying a lot of pore space; the solution permeates and diffuses through the hydrogel network, and the ion diffusion response time is relatively slow. A low pH stimulates hydrogels to shrink, and IOPCHs films internally maintain the porous structure of spherical hollows; the movement of the solution is mainly capillary diffusion through the holes. Assuming that the lateral expansion is small after the pH value increases, the pore space is filled with the expanded hydrogel, the porous structure of the IOPCHs extrudes and deforms, and the solution mainly permeates and undergoes mass transfer along the hydrogel network. In short, in alkaline conditions, the hydrogel is stimulated to expand, the pore diameter is small, and the solution ions mainly permeate and transfer mass along the hydrogel cell walls; in acidic conditions, hydrogels shrink, the pores are larger, and the solution ions mainly diffuse and undergo mass transfer along the pores (the IOPCH membrane is fixed on the substrate, thus limiting the lateral expansion).^122^ Therefore, high porosity can also improve the color change rate, and at the same time, the color change response time of a hydrogel at low pH is shorter than that of one at high pH.

Because OPCs have periodic arrangements, they can be analyzed as multilayer films with superimposed, two-dimensional (2D) porous crystal-structure single-layer films. According to Langmuir–Blodgett theory, IOPCH intramembranous structural units can be regarded as a combination of random CFC and HC crystal pore structures. Therefore, they can be analyzed using Bragg's optical law as follows:^123^

1 *λ*_max_ = 1.633 (*d*/*m*) (*D*/*D*_0_) (*n*_a_^2^ − sin *θ*^2^)^1/2^,

where *λ*_max_ is the wavelength at the maximum Bragg diffraction peak, *D* is the diameter of the inner hole of the hydrogel, *m* is the order of Bragg diffraction, *d* is the lattice constant, *D*/*D*_0_ represents the expansion degree of the hydrogel (*D* and *D*_0_ are the diameters of the inner hole of the hydrogel in the equilibrium state and the reference state under certain conditions, respectively), *n*_a_ is the average refractive index of the porous hydrogel to light, and *θ* is the included angle between the incident light and the diffraction crystal plane. According to this equation, when the environmental pH changes, the swelling degree of the hydrogel changes, and the diffraction peak of incident light shifts accordingly.

Based on pH stimulation, the optical diffraction peak position and the corresponding structural color of the IOPCHs films changed obviously. IOPCHs membranes contain weak acid (such as the carboxyl group) or weak base (amino group) pH-sensitive polyelectrolyte hydrogels; a change in the external pH will shift the acid–base dissociation balance of hydrogel molecules, and the molecular chains will reunite or disperse, resulting in the expansion or contraction of the hydrogel volume.^124^ This transfer of internal structure changes the lattice parameters of the internal holes of the IOPCH film. It can be seen that a change in the lattice constant, *d*, is the main reason for the obvious difference in the *λ*_max_ and the structural color under the same IOPCH film thickness.^125^ Conversely, the change in the environmental pH value can also be semi-quantitatively or quantitatively analyzed by directly observing the color change of the films with the naked eye or by testing the diffraction spectrum.

By adding buffer with a pH lower than 5, the pH value of the membrane can be reduced, the contraction of the membrane can be promoted, and the *λ*_max_ of the membrane can be blueshifted. The larger the pores, the greater the change in the *λ*_max_ value. Generally speaking, without passing through an epoxy filter, an excessive strong UV light will leads to serious polymerization reaction with a high crosslinking density and irregular delamination in the hydrogel, eventually cause narrow pores and difficult liquid mass transfer, which is not conducive to shortening the response time.

### 3.4. Principle of sol–gel transition discoloration

There is also a kind of pH-responsive IOPCHs that can reveal the mutual transformations of the sol and gel states, which change with the pH value. After dissociation or hydrolysis, pH-sensitive groups further destroy hydrogen bonds in the hydrogel, eventually causing changes in its internal network structure. Zhang *et al.*^126^ prepared a CS-based dynamic hydrogel with CS and functionalized polyethylene glycol as the main components. The two hydroxyl groups at the end of the polyethylene glycol chain were functionalized by benzaldehyde, which crosslinked the CS by a reversible Schiff base bond, and the hydrogel underwent a sol–gel transformation to generate a dynamic hydrogel. Because the aromatic Schiff base is sensitive to pH stimulation, it can trigger a shift in the Schiff base equilibrium and control the liquefaction or polymerization of the hydrogel. In the field of dynamic covalent chemistry, hydrogels that undergo gel–sol transitions driven by the environmental pH include borates, hydrazones, and imines.

#### 3.4.1 Borate esters

Some diols can be complexed with boric acid to form reversible borate, and the sol–gel characteristics of this borate complex are significantly related to changes in pH.^127^ For example, Wang *et al.*^128^ prepared a DN hydrogel with a pH response by using the crosslinking characteristic of the 1,3-cis hydroxyl group of the natural polysaccharide hydroxypropyl guar gum (HPG) and sodium borate (Na_2_B_4_O_7_·10H_2_O). In the lower pH range, DN hydrogel crosslinked to form a denser HPG network, and the swelling rate decreased. Conversely, at high pH, the crosslinking of the HPG hydrogel network was inhibited, and the swelling rate increased because of a decrease in B(OH)_4_^−^.

#### 3.4.2 Acylhydrazones

Acylhydrazone bonds are usually stable in neutral and alkaline conditions, but hydrolysis and cleavage are triggered in acidic conditions. pH-responsive hydrogels synthesized with this characteristic can realize the mutual transformation between the sol and the gel.^129^ Among these, hydrazone bonds formed by the reaction of hydrazide with a ketocarbonyl or aldehyde group under neutral or slightly acidic conditions are the most common. For example, Guo *et al.*^130^ generated hydrazone hydrogels using the reaction between the keto polymer diacetone acrylamide and adipic dihydrazide. The hydrazone bond can be easily hydrolyzed into sol in an acidic medium, and the gel can be quickly formed after the pH value is adjusted to above 5.0.

#### 3.4.3 Imines

Imine bonds are easy to hydrolyze in acidic conditions, and the gel changes into a sol state with a color change reaction.^131^ Yu *et al.*^132^ used the fact that imine bonds are easy to hydrolyze in acidic conditions and found that this kind of imine hydrogel can produce a sol–gel color change response based on the difference in the pH environments between cancerous tissue and normal tissue. Tao *et al.*^133^ used a Schiff base reaction between polyethylene glycol (PEG) and pentaerythritol tetra (3-mercaptopropionic acid) ester (PETMP)-allyl urea (AU) to synthesize a PETMP-AU-PEG hydrogel which was highly sensitive to pH changes.

#### 3.4.4 Other influencing factors

The swelling and shrinkage mechanisms of weak electrolyte hydrogels are extremely complex, and the existing models are mainly divided into a three-phase model and a multi-effect-coupling response model.^134^ In the three-phase model, the three phases are the solvent phase, the free ion phase, and the polymer network phase. This model uses constitutive relations, momentum conservation, and mass conservation to analyze the swelling–shrinkage behavior of hydrogels in different conditions.^135^ The electrochemical and mechanical equations coupled *via* hydration are collectively called the multi-effect-coupled pH response model.^136^ This model is based on geometrically nonlinear finite deformation theory and generally consists of highly nonlinear coupling parts and ordinary differential equations in the coupling domain, which can better study the large deformation behavior of pH-sensitive hydrogels and the local swelling changes in hydrogels.^137,138^

Environmental factors such as temperature, solvent composition, and ionic strength will also affect the characteristics of hydrogels.^139^ Li *et al.*^140^ studied differences in the pH sensitivity of γ-polyglutamic acid hydrogels in two different solutions, NaCl and CaCl_2_. The gel pH sensitivity of hydrogels in CaCl_2_ solutions is very weak, and the swelling rate of hydrogels in solutions at pH 1–7 varies from 2.5 to 5 g g^−1^. The gel pH sensitivity in the NaCl solution in the same pH range is very strong, and the swelling rate varies from 5 to 28 g g^−1^. Similarly, for example, Yan *et al.*^141^ found that the molecular weight of hydrogels can also lead to a difference in pH sensitivity. After the crosslinking of poly-l-glutamic acid/CS, when the pH is less than 7, the network structure gel with a low relative molecular weight is loose. In acidic conditions, water molecules mainly diffuse into the gel by swelling, and small molecular hydrogels are more sensitive to the pH of the solution. With a gradual increase in the pH value in alkaline conditions, the carboxyl groups on the hydrogel segments begin to ionize, and more COO^−^ ions are generated compared with gels with a high molecular weight. Therefore, macromolecular hydrogels are more sensitive to a solution's pH.

**Fig. 12:**
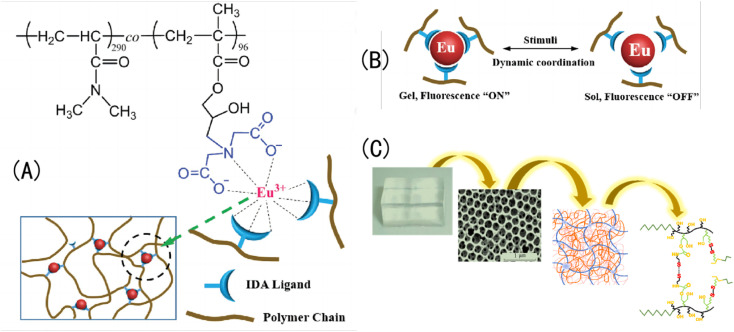
(A) Chemical structures of Eu–IDA; (B) schematic illustration of Eu–IDA sol–gel transition driven by dynamic metal–ligand coordination;^143^ reproduced from ref. 143 with permission from Wiley-Blackwell, copyright 2018. (C) Analytical schematic diagram of the microstructure of an IOPCH film.

Otherwise, the dynamic characteristics of metal coordination of IOPCHs membranes can also make hydrogels reversibly form and separate the metal coordination under different pH stimuli, and they show different colors by forming different metal-chelating bonds.^142^ For example, Weng *et al.*^143^ used iminodiacetate (IDA) as a ligand to bind Eu^3+^ ions, which can also be used as crosslinking agents to construct hydrogel networks. When the pH of a solution is acidic, Eu^3+^ ions dissociate from IDA ligands; when the pH of a solution is alkaline, the coordination of Eu–IDA can be restored, and the reverse reversible transformation occurs, as shown in Fig. 12.

### 3.5. Principle of thickness change discoloration

According to research work by Tanaka *et al.*,^144^ there is a relationship between the response time of ions in an IOPCH membrane and the volume of hydrogels; formula (2) shows that the response time required for the expansion–contraction of an IOPCH membrane stimulated by an external acid or alkali is directly proportional to the square of its thickness and inversely proportional to the effective diffusion rate of the ions in polyelectrolyte hydrogels or water; thus, multilayer IOPCH membrane usually have longer response times. Therefore, reducing the membrane thickness and volume fraction of polyelectrolyte gels as much as possible can effectively shorten the response time of an IOPCH membrane.

The following is an example of an equation for calculating the response time:2
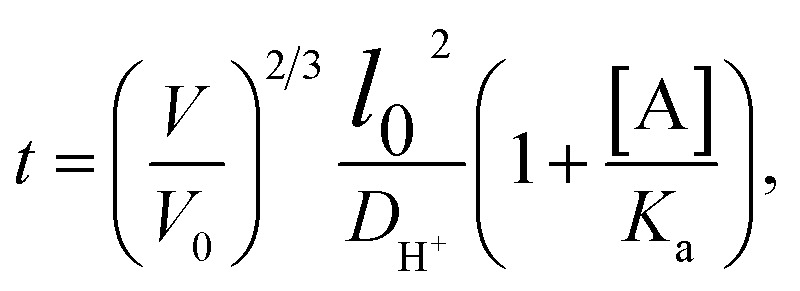


**Fig. 13:**
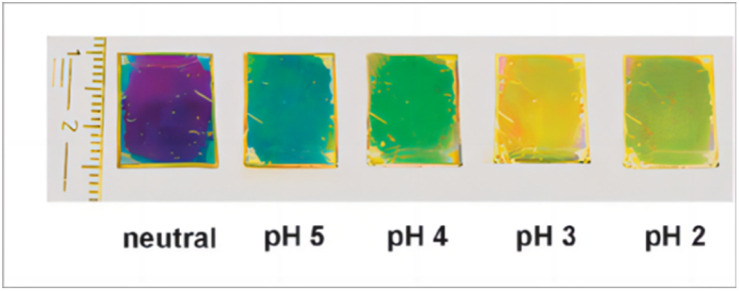
Photograph of color changes in single-layer ultrathin IOPCH film at different pH values.^146^ Reproduced from ref. 146 with permission from Royal Society of Chemistry, copyright 2012.

In formula (2), *V* and *V*_0_ are the final volume and initial volume of the IOPCH film, respectively; *L*_0_ is the thickness of the IOPCH film; *D*_H^+^_ is the effective diffusion rate of ions in polyelectrolyte IOPCH or water; [A] is the concentration of carboxyl groups in the IOPCH film; and *K*_a_ is the dissociation constant of the carboxyl group in water. Li *et al.*^145^ prepared a pH-responsive single-layer polyelectrolyte IOPCH film with a thickness of 470 nm; this film could reach a response equilibrium state in only 6 s. Although it only has a submicron thickness, it still has good color visibility to the naked eye, as shown in Fig. 13.

A critical analysis of the swelling process reveals two underlying molecular processes: the penetration of the solvent molecules into void spaces in the network and the subsequent relaxation of the network segments. According to the research results of Dengre,^147^ the diffusion coefficient, *D*, of water in an IOPCH membrane can be obtained using the following formula:^148^

3 *F* = 4[*Dt*]^0.5^/[π*r*^2^] − π[*Dt*]/[π*r*^2^] − [π/3][*Dt*]^1.5^/[π*r*^2^]

where *F* denotes the amount of the solvent fraction at response time *t*, *k* and *n* are constants, and *r* is the thickness radius of the hydrogel.

In different pH solutions, the *n* values of all kinds of hydrogels are between 0.5 and 1, which shows that solutions with different pH values have no obvious influence on the initial diffusion behavior of hydrogels, and they all belong to the non-Fickian diffusion mode. It also shows that the diffusion rate of water molecules is equivalent to the relaxation rate of gel network macromolecules in the initial stage of swelling in IOPCH membrane.^92,96^4
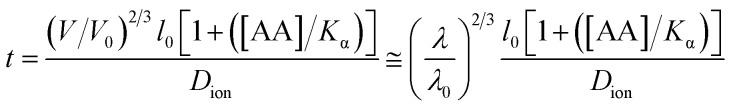
Here, *l*_0_, [AA], *K*_α_, and *D*_ion_ respectively denote the hydrogel thickness, the concentration within the hydrogel, the acid dissociation constant, and the diffusion constant of proton.

The ionization degree of –COO^−^ generated *via* hydrolysis in strong alkali solutions is determined by the p*K*_a_ and pH values of the solution. In an acidic environment, –COO^−^ on the skeleton of a hydrogel combines with H^+^ in the solution to generate carboxylic acid, which leads to a decrease in the osmotic pressure inside the hydrogel and a corresponding reduction in the volume of the hydrogel, until it reaches equilibrium.^92^ At the same time, the pores between colloids in the IOPCH membrane expand, and the reflection peak is blueshifted. In alkaline conditions, given the mass transfer of solutes through the swollen hydrogel, the diffusion is much slower, and this hindered diffusion is reflected by t_2_, which is different from the rapid diffusion t_1_, because there are still some pore spaces inside the hydrogel.^98^ When the pH of the solution is increased to 9 or even greater, the ionization of carboxyl groups on the hydrogel is complete, and the hydrogel film expands, resulting in a reduction in the colloid volume between reverse opal structures and a redshift of the reflection peak of the film. Further increasing the pH will only increase the ionic strength of the solution, but will lead to the contraction of the hydrogel. Given the mass transfer of solutes through the swollen gel, the diffusion is much slower, and this hindered diffusion is reflected by t_2_, which is different from the rapid diffusion t_1_, because there are still some pore spaces in the hydrogel.^149^

The above shows that the principles of color-changing reactions vary greatly dependent on the different components of hydrogels. At the same time, the main factors that determine the response performance of a color-changing film are as follows: the material composition of the film, the synthesis conditions (such as ultraviolet irradiation time, light intensity, temperature, *etc.*), the appropriate thickness, and the regularity of the porosity of the film. These are the key factors for increasing the color-changing response performance of the membrane, reducing the response time, optimizing the pore composition in the membrane, and changing the physical and chemical properties of the membrane surface. Next, several specific directions for improving membrane performance are summarized based on the preparation methods.

## 4. Discussion

**Fig. 14:**
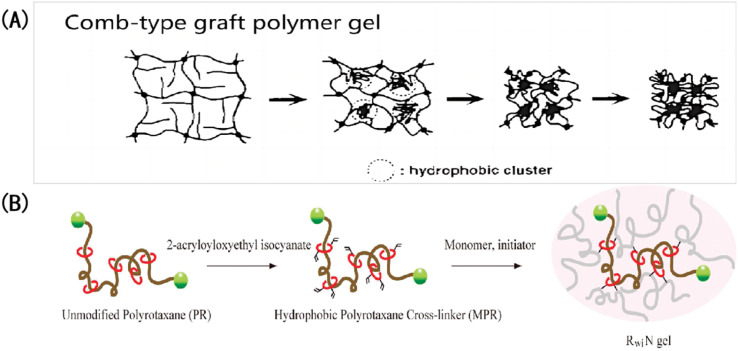
Accelerating the response of IOPCHs membranes with hydrophobic modification: (A) structural shrinkage mechanism of hydrophobic-grafted poly (*N*-isopropylacrylamide) gel induced by the environment;^153^ reproduced from ref. 153 with permission from Springer Nature, copyright 1995. (B) Schematic diagram of the application of hydrophobic crosslinking agent polyrotaxane to modify gel in a dimethyl sulfoxide solution.^152^ Reproduced from ref. 152 with permission from American Chemical Society, copyright 2010.

The above pH-IOPCHs films have some shortcomings, such as a relatively long response time, incident light angle dependence, and a lack of further complex structures. If these shortcomings can be improved on, the development of advanced color-changing optical equipment can be promoted. Based on our analysis of the discoloration principle of hydrogels, the main technical improvement directions are as follows:

(1) Because the band gap position or structural color of pH-IOPCHs films can dynamically change in the visible light range with external stimuli, adjusting the hydrophilicity and hydrophobicity of a membrane surface can effectively regulate the mass transfer of the solution, and an appropriate film surface roughness can accelerate the swelling balance of the gel system and improve the response rate.^150^ Because surface hydrogen bonding and hydrophobic interactions affect the mass transfer process between ions in the solutions and the gel network, generally speaking, the stronger the surface hydrophobicity, the more difficult the mass transfer. Yin *et al.*^151^ prepared a pH-IOPCH membrane that can change its surface roughness, and its response speed can be adjusted based on different hydrophilic and hydrophobic properties of the membrane surface. Takeoka *et al.*^152^ studied the influence of hydrophilic and hydrophobic crosslinking agents on the swelling behavior of a poly (isopropylacrylamide) gel network. They found that the gel modified by hydrophobicity could reach its deswelling equilibrium more quickly, mainly because hydrophobic chains gathered during the gel contraction, forming large crystal nuclei and water-rich regions, which communicated with each other to form water channels. Thus, water could be discharged from the channels more freely, and the gel contraction was accelerated, as shown in Fig. 14.

(2) The mass transfer speed of a solution in a membrane also depends on the internal structure of the membrane. With the same membrane thickness and pH environment, the crosslinking density, pore spacing, and pore size parameters of the membrane play a decisive role in the response speed.^154^ In the synthesis process, when ultraviolet irradiation is too strong or the irradiation time is too long, the hydrogel has a high crosslinking density, smaller interconnected pores, a larger hydrogel content, and a smaller expansion–contraction degree in the same pH environment.^155^ Therefore, optimizing the exposure time, intensity, and wavelength of the ultraviolet lamp reasonably is one of the keys to realize the rapid-response of pH-IOPCHs films.

(3) The influence of changes in the p*K*_a_ value of a hydrogel on its expansion coefficient at different pH values plays a decisive role in its color changes. The complete balance expression of the influencing factors of the Flory–Huggins mixed term can be used to explain the relationship between the color redshift term of the IOPCH membrane in an acid–base solution and environmental factors, that is, the composition of the polymer network, its porous structure, elastic contraction and expansion parameters, changes in the pH value, and the comprehensive contribution of the solution's ion concentration to membrane osmotic pressure, as shown in the following formula:^156^5
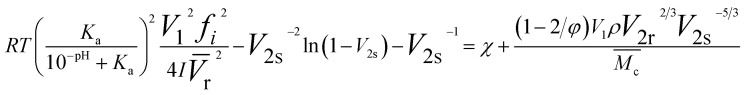
where *R* is the atmospheric gas constant; *T* is the ambient temperature; *K*_a_ is the acid–base equilibrium constant in a neutral solution; the polymer volume fraction of the hydrogel is *V*_2r_; the polymer volume fraction of the swollen gel is *V*_2s_; *φ* is the number of branches in a hydrogel crosslinking network site; π_ion_ is the pressure difference in exchanged ions inside and outside the hydrogel; *V*_1_ is the molar volume of the solvent; and *f*_*i*_ is the molar fraction of ionic units in the gel system. (*V̄*_r_) is the average molar volume of the pore repeating unit in the polymer, I is the ionic strength in the swelling medium, *χ* is the swelling parameter in the polymer–solvent interaction, *ρ* is the density of the polymer network, and (*M*_c̄_) is the average molecular weight of the gel crosslinked network.

A volume change will lead to a deviation in refractive index, and then, the color will be different. The formula more intuitively reflects the relationship between environmental temperature, ionic strength, environmental pH, the polymer expansion coefficient, polymer volume change, and other related parameters.

**Fig. 15:**
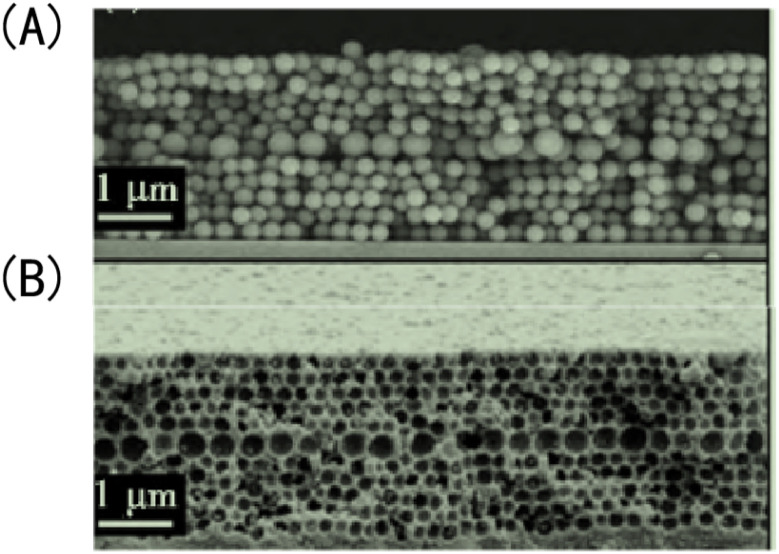
Scanning electron microscope image of IOPCH films containing a macropore defect layer: (A) opal structure; (B) anti-opal structure.^159^ Reproduced from ref. 159 with permission from Royal Society of Chemistry, copyright 2011.

(4) Increasing the porosity or pore size of pH-responsive hydrogels is another effective way to shorten the response time of IOPCH membrane because they are usually porous structures formed by removing the opal structure template from the functional hydrogel *via* dissolution or chemical etching.^157^ If there is a large number of continuous holes in the IOPCH membrane compared with a homogeneous hydrogel with the same composition and quality, this provides a large number of water paths for the solution ions to diffuse through by capillary action inside the gel, which improves the effective diffusion rate of the solution ions and effectively shortens the response time.

(5) Reducing the thickness reasonable can effectively balance the response rate and service life of hydrogels. For example, Lee *et al.*^94^ prepared a 23 μm thick pH-IOPCH film made of modified PS-b-QP2VP using the colloidal crystal template method, and its response time was shortened to 20 min. Because the crosslinking density was small, the permeability was reduced, and the sensitivity of the IOPCH membrane was also improved to some extent. At the same time, the proper amount of HEMA and polymerization duration provided an optimized IOPCH membrane with improved mechanical stability and a long service life (more than 6 months), without prolonging the response time or deteriorating the pH response performance. In brief, a smaller crosslinking density, larger pore size, and relatively less hydrogel content can increase the diffusion paths of ions inside the hydrogel, improving the effective diffusion rate and greatly shortening the response time of the IOPCH membrane.^158^

(6) The usual way to optimize porous structures inside membranes is to optimize the synthesis parameters of the IOPCH membrane, which is highly dependent on the physical and chemical properties of its hydrogel. Griffete *et al.*^159^ found that introducing a macroporous structural defect layer to an IOPCH membrane can significantly improve its response rate, as shown in Fig. 15. By testing the properties of IOPCH films with and without defect layers of the same thickness, they showed that IOPCH film with defect layers have greater porosity, which affects the initial periodic uniform porous structure. Such a local defect state accelerates the ion capillary diffusion rate of the solution and shows a faster response rate (the fastest response time is less than 10 s). However, it is still difficult to prepare defect structures with good control because of the inherent microstructure defects in the self-assembly process of colloidal crystals.

(7) By modifying the surface of a hydrogel, such as by enhancing its hydrophobicity and increasing its mechanical strength, its service life can be improved. At present, high-toughness hydrogels mainly include the following: interpenetrating-DN-structure IOPCHs, inorganic/organic nanocomposite IOPCHs, hydrophobic-interaction-crosslinked IOPCHs, *etc.*^160^ Among these, an interpenetrating-DN-structure IOPCH is a dense network structure copolymer formed by intertwining rigid and flexible polymer networks. In the process of stress, the rigid network first breaks to absorb energy, and the strength of the hydrogel is improved by a “sacrificial bond”, while the flexible network is loose to better realize energy transfer and dispersion.^161,162^

**Fig. 16:**
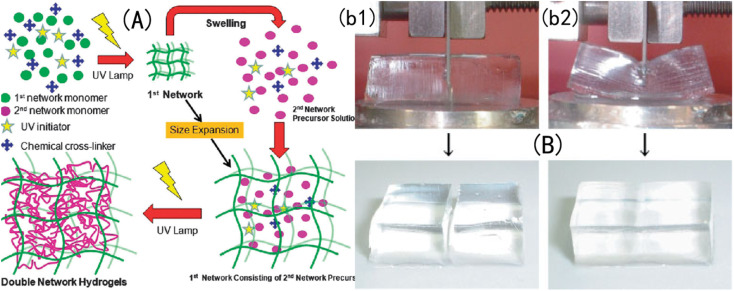
Structural diagram of (A) a DN-structured IOPCH network film^166^ reproduced from ref. 166 with permission from Royal Society of Chemistry, copyright 2015. (B) Where the strength of the DN-structured IOPCH film (b2) is significantly higher than that of a single-network hydrogel (b1).^163^ Reproduced from ref. 163 with permission from Royal Society of Chemistry, copyright 2003.

A DN-structured IOPCH was first proposed and successfully prepared by Gong *et al.*,^163^ as shown in Fig. 16, in which the first network is poly-2-acrylamide-2-methylpropanesulfonic acid (PAMPS) gel and the second network is PAAm. Zheng *et al.*^164^ used agar with strong intramolecular hydrogen bonding as the first network and neutral PMMA as the second network to prepare an agar/PMMA interpenetrating DN gel with good mechanical properties and fatigue resistance. The tensile strength at break can reach 10 MPa, the elastic modulus can reach 1 MPa, and the breaking energy can reach 100–1000 J m^−1^. Nanocomposite hydrogels are prepared by dispersing high-modulus nanoparticles into a polymer matrix *via* physical or chemical crosslinking, which toughens the hydrogels.^165^

## 5. Conclusions

**Fig. 17:**
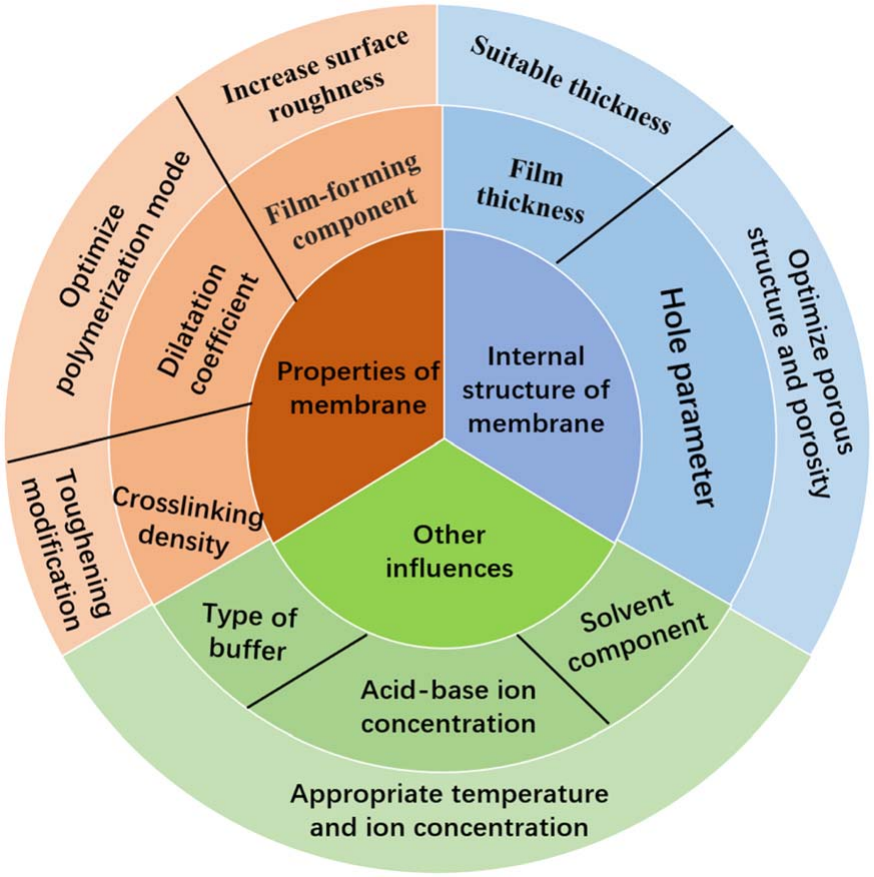
Schematic diagram of factors affecting the performance of IOPCHs films and improvement methods.

Because of its unique 3D network porous structure and good optical and mechanical properties, IOPCH film shows great potential for application in the fields of structural coloration, sensors, photocatalysis, and biomedicine. At the same time, designing and constructing polymer thin-film materials with optically variable lattice porous structures inside colloids has strong development prospects. With ongoing research, more and more materials are being used to prepare pH-IOPCHs membranes, and the process is constantly being optimized and innovated, with the quality of the membranes becoming better and better. However, the performance of pH-IOPCH still faces great challenges and needs to be improved, as shown in Fig. 17. Based on the above comprehensive discussion of the preparation methods, discoloration principle, and influencing factors of film performance, the specific technical bottlenecks and solutions are summarized as follows:

(1) Many kinds of OPCs with strong functions can be prepared using the template method, but during the prepolymer filling process, OPC templates may be destroyed by the prepolymers, and the limitations of template materials make it difficult to prepare large-area IOPCH film, mainly because it is difficult to assemble large-area OPC templates. If large-area and defect-free OPC template assembly can be created, large-area IOPCH film that meet practical application requirements can be prepared, but the larger the template area, the easier it is to produce defects. Many kinds of IOPCHs films with strong functions can be prepared with the template method; however, during the prepolymer filling process, OPC templates may be corrupted. Therefore, it is particularly important to find new synthesis methods, such as combining the template method with the spraying method and the continuous 3D printing method.

(2) At present, only SiO_2_, PS, PMMA, and a few other materials are commonly used as template microspheres to prepare IOPCHs films; moreover, the films can only be removed using traditional methods, such as acids and alkalis, high temperatures, and acetone. Acid hydrolysis corrosion removal damages the membrane material's body; high-temperature removal can easily lead to the collapse of the porous structure of the inverse opal; and acetone, as an organic solvent, can easily affect organic membrane materials. Therefore, finding a microsphere template material that can be removed without damaging the membrane matrix in mild conditions has gradually become the focus of research. Designing reasonable film-forming conditions and controlling the preparation process are the keys to avoiding defects in IOPCH film structures.

(3) In addition, ultraviolet irradiation and high-temperature heating may also cause cracks and defects in IOPCH film. The internal forces of organic colloids can also lead to incompleteness in the membrane, which will affect its optical properties. For example, when an ionic AA monomer is introduced to a charged QP2VP layer when an IOPCH film is prepared using the copolymerization method, the monomer distribution is often uneven because of interactions between internal ionic charges, forming an irregular “rainbow color” on the surface, which leads to inevitable structural defects.

(4) In theory, the high porosity, uniformity, and easy regulation of pores in IOPCH membrane can be applied to many fields, but in fact, the applications of pH-IOPCHs membranes are scarce at present, which may be due to the small thickness of the assembled crystal template, leading to membrane material that is too thin and mechanical strength that cannot withstand mechanical damage from the natural environment. This limits their application. Although the thickness should be as small as possible, which is the most effective way to improve the response time of IOPCHs films, a too-small thickness will reduce the membrane's mechanical properties and affect its service life. Finding the optimal thickness solution between the response time and the mechanical properties of the membrane in certain conditions is also one of the key points to be addressed.

(5) Traditional polymer hydrogel materials are formed *via* the simple covalent bonding of polymer monomers or natural polymers. Because of the uneven distribution of covalent crosslinked link networks, hydrogels are easily damaged by external forces during use. Therefore, it is very important to design multifunctional high-toughness hydrogels to enhance their service life. Moreover, pH-IOPCHs membranes can be recycled for half a year at the longest, and their reusability needs to be improved. Repeated cyclic discoloration can easily lead to reductions in the environmental tolerance of IOPCHs membranes or even damage the porous structure in the membrane, resulting in problems such as insufficient color or an inability to change color. Therefore, the durability of the membrane's porous structure can be ensured by modifying the hydrogel network *via* plasticization, thus improving the service life of the membranes. It is of great importance to improve the performance of pH-IOPCHs and develop their potential application in real-time analyses, online detection, environmental stealth, and other fields.

## Data availability

The data that support the findings of this study are available from the corresponding author upon reasonable request.

## Author contributions

Hu Wei, conceptualization, paper writing; Chen Changbing, analysis of article structure and error proofreading; Yang Dafeng, collecting data and providing financial support. Hu Wei's writing, first draft preparation. Yang Dafeng and Chen Changbing's writing, review and proofreading.

## Conflicts of interest

The authors declare no conflict of interest.

## Supplementary Material
